# Automation of antimicrobial activity screening

**DOI:** 10.1186/s13568-016-0191-2

**Published:** 2016-03-12

**Authors:** Samuel P. Forry, Megan C. Madonna, Daneli López-Pérez, Nancy J. Lin, Madeleine D. Pasco

**Affiliations:** Biosystems and Biomaterials Division, National Institutes of Standard and Technology, Gaithersburg, MD 20899 USA; Center for Drug Evaluation and Research, Food and Drug Administration, Silver Spring, MD 20993 USA

**Keywords:** Antimicrobial activity screening, Automation, Performance assessment

## Abstract

Manual and automated methods were compared for routine screening of compounds for antimicrobial activity. Automation generally accelerated assays and required less user intervention while producing comparable results. Automated protocols were validated for planktonic, biofilm, and agar cultures of the oral microbe *Streptococcus mutans* that is commonly associated with tooth decay. Toxicity assays for the known antimicrobial compound cetylpyridinium chloride (CPC) were validated against planktonic, biofilm forming, and 24 h biofilm culture conditions, and several commonly reported toxicity/antimicrobial activity measures were evaluated: the 50 % inhibitory concentration (IC50), the minimum inhibitory concentration (MIC), and the minimum bactericidal concentration (MBC). Using automated methods, three halide salts of cetylpyridinium (CPC, CPB, CPI) were rapidly screened with no detectable effect of the counter ion on antimicrobial activity.

**Electronic supplementary material:**

The online version of this article (doi:10.1186/s13568-016-0191-2) contains supplementary material, which is available to authorized users.

## Introduction

The automation of biological assays promises to improve reproducibility by minimizing ‘human error’ and to increase experimental throughput by tirelessly repeating standardized operations (Felder 1998; Tomar 1999; Sarkozi et al. 2003). In research environments, automated systems have amplified the speed and accuracy with which investigators can produce reliable and reproducible results (Sarkozi et al. 2003; Linshiz et al. 2012). In contract laboratories, commercially available liquid handling robots perform many labor intensive, repetitive, or hazardous tasks such as pipetting reagents, extracting DNA, manipulating pathogens, and performing routine cell culture (Felder 1998; Young 2000; Armstrong 2012). Investigations that take advantage of automation often benefit from standardized protocols, improved inter-laboratory repeatability, reduced reliance on manual operations, higher throughput and statistical power, and increased productivity.

Using mammalian cell culture lines, automation is routinely used to achieve high-throughput toxicity screens of novel chemical compounds, either to detect adverse off-target effects of potential therapeutics, or to measure desired activities (e.g., killing cancer cells) (Alley et al. 1988; Shoemaker 2006; Kim et al. 2013). Standardized procedures, quantitative endpoint assays, and generally well-understood toxicity mechanisms contribute to the reliability of these cell-based toxicity assays. In contrast, the screening of bacterial cultures for toxicity from new compounds (e.g., to evaluate potential new antimicrobials) is much less commonplace and typically relies on manual laboratory operations (Wilson 1996; Sabater et al. 2007; Valgas et al. 2007). Many different procedures and measurement strategies have been developed for the evaluation of antimicrobial activity (i.e., bacterial toxicity), with varying degrees of accuracy, reproducibility, expense, and throughput (Sabater et al. 2007; Wilson 1996; Xiao et al. 2008; Haps et al. 2008; Biswas and Biswas 2011; Bhattacharya et al. 2003; Pallanza et al. 1984; Harrison et al. 2004).

Unlike mammalian cell toxicity screening, microbial cultures can exhibit dramatically different susceptibilities to antimicrobial compounds depending on culture conditions and phenotype (Marsh 2004; Harrison et al. 2004; Sabater et al. 2007). For instance, bacterial biofilms typically exhibit significantly reduced sensitivity as compared to planktonic cultures, though the exact relationship varies with the specific bacterial strain, antimicrobial compound, and culture conditions (Pallanza et al. 1984; Wilson 1996; Marsh 2004; Harrison et al. 2004; Salli and Ouwehand 2015). Bacteria in the environment frequently transition between various phenotypes (e.g., planktonic and biofilm), and it is often unclear which culture conditions will accurately reflect environmental exposure (Wilson 1996; Baehni and Takeuchi 2003; Harrison et al. 2004; Sabater et al. 2007; Valgas et al. 2007).

The bacteria in the oral environment are well studied, accessible, and similar to many other naturally occurring microbial ecologies in that they typically incorporate many different strains and phenotypes in dynamic competition for limited nutrients (Biswas and Biswas 2011; Marsh 2004, 2005; Gibbons and Houte 1975; Kuramitsu et al. 2007; Cvitkovitch et al. 2003; Kolenbrander 2000; Scannapieco 1994; Liljemark and Bloomquist 1996; Loesche 1986; Salli and Ouwehand 2015). *Streptococcus mutans* has been widely studied as a model organism that produces cultures representative of dental plaque and illustrative of processes contributing to dental decay (Hamada and Slade 1980; Pallanza et al. 1984; Loesche 1986; Scannapieco 1994; Liljemark and Bloomquist 1996; Wilson 1996; Kolenbrander 2000; Bhattacharya et al. 2003; Cvitkovitch et al. 2003; Xiao et al. 2008; Biswas and Biswas 2011; Cornejo et al. 2013; Kitagawa et al. 2014; Salli and Ouwehand 2015).

We report here an evaluation of automated liquid handling instrumentation for routine, high-throughput screening of emerging antimicrobial compounds against *S. mutans*. Automated methods were developed for the evaluation of bacterial growth under planktonic, biofilm forming, and 24 h biofilm growth conditions. For each, antimicrobial activity was evaluated by measuring the half maximal inhibitory concentration (IC50), the minimum inhibitory concentration (MIC) and the minimum bactericidal concentration (MBC). The results from automated assays of a known antimicrobial compound [cetylpyridinium chloride (CPC)] compared favorably with results from manual assays, though the automated methods were considerably faster. Using the faster automated methods, three cetylpyridinium salts with different halide counter ions were screened for antimicrobial activity.

## Materials and methods^1^

### Materials

*Streptococcus mutans* (UA159) were obtained from ATCC (700610). Todd Hewitt Broth (THB) powder and agar were obtained from BD. Yeast extract, dextrose, and glycerol were obtained from Sigma. 96-well (Falcon tissue culture polystyrene) and 1-well (OmniTray) plates were obtained from Corning Life Sciences and Thermo Scientific, respectively. The automated liquid handler robot (Biomek FX), as well as regular (AP96 P250) and conductive (Span8 P250) pipette tips, were obtained from Beckman Coulter. XTT (2,3-bis(2-methoxy-4-nitro-5-sulfophenyl)-S-[(phenylamino)carbonyl]-2*H*-tetrazolium hydroxide) was obtained from Fluka. Sterile phosphate buffered saline (PBS) was obtained from Gibco. Phenazine methosulfate (PMS) and cetylpyrininium bromide (CPB, 384.19 g/mol) were obtained from Acros Organics. Cetylpyrininium iodide (CPI, 431.79 g/mol) and chloride (CPC, 339.99 g/mol) were obtained from City Chemical, LLC and MP Biomedicals, Inc., respectively.

### Media formulation

THB was reconstituted at 30 g/L in distilled water (EasyPure II). Biofilm-forming media (BFM) was comprised of 7.5 g/L THB, 1.25 g/L yeast extract, and 10.25 g/L dextrose in distilled water. Agar was prepared by dissolving 15 g/L agar in THB. All solutions were autoclaved (Tomy ES-315; 120 °C for 20 min) prior to use.

### Protocols and automation

All assays were performed in standard multiwell plates to facilitate throughput and automation. Laboratory operations that involved manipulating liquids (e.g., inoculating suspended cells, dispensing media, diluting and distributing solutions of dissolved chemicals) and timed periods of incubation (e.g., overnight growth, media exchange, XTT conversion) were particularly amenable to automation. For this investigation, manual assays were performed with Eppendorf single barrel pipettes, while automated assays were performed using the Biomek FX automated liquid handler. Positive and negative control conditions were included on every antimicrobial activity assay plate (the plate layout is available in the Additional file 1: Figure S1). Data analysis and visualization were performed using R(R Core Team 2014) and several freely-available R packages: ggplot2, reshape, beeswarm, ggplot2bdc, xlsx (Wickham 2007, 2009; Eklund 2013; Connelly 2014; Dragulescu 2014). All raw data and analysis code are publically available (Forry 2015).

### Culture methods

#### Stock cultures

Fresh planktonic *S. mutans* cultures were initiated in THB from 25 % glycerol stock stored at −80 °C, or from streaked agar plates (stored at 4 °C for up to 8 weeks). These starter cultures were incubated (37 °C and 5 % CO_2_, unless otherwise indicated) overnight before being diluted to inoculate cultures in 96-well plates at an optical density (OD, measured at 630 nm) of 0.008 for 160 µL (OD of ≈0.02 for 1 cm path length). Planktonic and biofilm cultures were inoculated similarly, but utilizing different media compositions (THB and BFM, respectively).

#### Planktonic assays

For planktonic assays, cells from the overnight culture were inoculated into THB containing defined dilutions of antimicrobial compounds (i.e., CPC, CPB, CPI). After overnight incubation, growth was assessed by measuring increased turbidity, and 5 µL of the suspended cells were inoculated onto agar to evaluate colony formation.

#### Biofilm forming assays

Antimicrobial activity during biofilm formation was assessed by inoculating cells from the overnight stock cultures into BFM containing defined dilutions of antimicrobial compounds. After overnight (24 h) incubation, the media was replaced with BFM without antimicrobial compounds for 3 h before growth was assessed by measuring the biofilm metabolic activity. Subsequently, cells from the biofilm were dislodged by vigorous pipetting, and 5 µL of the resulting suspension were inoculated onto agar to evaluate colony formation.

#### 24 h biofilm assay

More mature biofilms were evaluated by inoculating cells from the overnight culture into BFM without antimicrobial compounds. After incubating 24 h, the media was replaced with fresh BFM containing defined dilutions of antimicrobial compounds for 3 h before the biofilm metabolic activity was measured. Subsequently, cells from the biofilm were dislodged by vigorous pipetting, and 5 µL of the resulting suspension were inoculated onto agar to evaluate colony formation.

### Culture analysis

#### Turbidity

The growth of planktonic cultures was monitored by measuring increased turbidity, as indicated by solution optical density (OD) at 630 nm on a plate reader (BioTek ELx808).

#### Metabolic activity

Biofilm metabolic activity (for both biofilm forming and 24 h biofilm conditions) was determined using the XTT reagent, as reported previously (Scudiero et al. 1988; Koban et al. 2012). After adding BFM for 3 h to refresh metabolic activity, the media was replaced with XTT solution (0.18 mg/ml XTT and 0.02 mg/ml PMS in PBS). Metabolic activity was monitored by measuring the conversion of XTT to a formazan derivative in solution. The derivative exhibited strong absorbance at 450 nm that was quantified on a plate reader. To correct for apparent absorption that was due to light scattering from the biofilm that coated the well bottom, the absorbance at 630 nm was subtracted. The background signal (e.g., from the presence of antimicrobial compounds) was also measured in negative control wells that contained no cells and was subtracted. The XTT solution was replaced with fresh BFM before evaluating colony formation.

#### Agar colony formation

Colony formation was evaluated by plating cells onto agar. For planktonic cultures 5 µL of the suspended cells were simply dispensed onto an agar plate. For biofilm cultures, adherent cells were first dislodged from the biofilm by vigorous pipetting. Using the automated liquid handler, pipette tips were positioned 1 mm above the bottom of 96-well plates in the center of the well, and 50 µL media was aspirated and dispensed 10 times at 200 µL/s. Finally, the pipette tips were lowered to touch the bottom of the well (height = 0 mm) before aspirating 5 µL for transfer to an agar plate.

For both planktonic and biofilm cultures, 5 µL of suspended cells from each well of an antimicrobial activity assay plate were inoculated onto the agar surface using the liquid-level sensing capability of the automated liquid handler. Briefly, agar was prepared in 1-well dishes with standard multi-well plate dimensions. Conductive pipette tips were used and the machine identified contact with the agar during each approach by the resulting change in tip capacitance. This established the proper height for dispensing suspended cells. On the agar, surface tension effectively immobilized droplets and prevented spreading, so the arrangement of discrete inocula retained the spatial organization of the 96-well source plate. The prepared agar plates were incubated 48 h to allow colony formation, and the presence or absence of growth (Boolean designation) was manually determined by visual inspection. The reported MBC was the lowest antimicrobial concentration that resulted no colony formation on the agar. An image of the resulting agar plate containing colonies is provided in the Additional file 1: Figure S1.

### Antimicrobial activity analysis

For each type of culture, the effect of antimicrobial compounds on microbial growth was quantified in three ways: IC50, MIC, and MBC. Assays were performed using twofold serial dilutions in antimicrobial concentration. For each antimicrobial concentration, at least four replicate wells were considered. Additional positive and negative controls were incorporated in every antimicrobial activity assay plate, including each antimicrobial compound concentration in the absence of bacteria, bacteria in the absence of antimicrobials, and media in the absence of bacteria or antimicrobials. The plate layout is provided in the Additional file 1: Figure S1.

#### IC50

The IC50 is commonly used to characterize toxic compounds by the midpoint of their effect on culture growth (Sebaugh 2011). This midpoint was calculated from a three-factor nonlinear fit to the experimental data in R using a logistic equation:

$$ Signal = \frac{{\left( {a - d} \right)}}{{1 + \left( {\frac{Concentration}{\text{IC50}}} \right)^{ - 30} }} + d $$

where *Signal* represented bacterial growth (turbidity or metabolic activity for planktonic or biofilm cultures, respectively), *a* and *d* were the plateau response for high and low antimicrobial concentrations, respectively, and *Concentration* and *IC50* (of the antimicrobial compound) were evaluated on a log scale. The exponent of −30 was manually chosen to simplify the fitting algorithm. Repeated antimicrobial assays (on different days) consistently produced IC50 values that varied by ≤30 %.

#### MIC

Another common metric, particularly for evaluating antimicrobial materials, is the MIC. The MIC was determined by identifying the lowest antimicrobial concentration that completely inhibited growth in all replicate wells. Because twofold dilutions of antimicrobial were considered, the expected precision of MIC determination was a factor of 2.

#### MBC

Finally, the MBC is the antimicrobial concentration that actually kills bacteria (instead of just preventing growth). MBC assessments require that samples from cultures be inoculated onto agar following exposure to the antimicrobial compounds. Colony formation on agar after 48 h of incubation can reveal residual surviving bacteria even when no growth was detected initially. The reported MBCs were the minimum antimicrobial concentrations for which no colony formation was observed by visual inspection. Because twofold dilutions of antimicrobial were considered, the expected precision of MBC determination was a factor of 2.

## Results

### Cross contamination during automated liquid handling

**Fig. 1:**
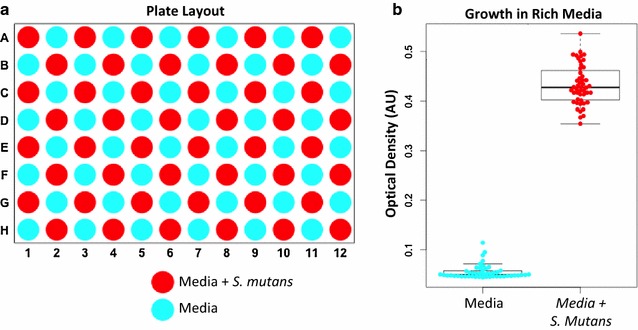
Multiwell plate cross-contamination. Into a 96-well plate uniformly filled with rich media (THB), *S. mutans* bacteria were inoculated into alternating wells using an automated liquid handling robot (**a**). After 24 h, planktonic growth was observed in all wells inoculated with bacteria, while no growth was observed in any negative control wells containing media alone (**b**)

Using an automated liquid handling instrument, the potential for contamination during plate preparation was evaluated by filling an entire 96-well plate with sterile, nutrient-rich media (THB) and then inoculating alternate wells with bacteria (Fig. 1). Planktonic growth was assessed by increased solution turbidity after 24 and 48 h of incubation, and the experiment was repeated twice. While all wells intentionally inoculated with bacteria exhibited growth, no bacteria were observed in any of the remaining wells. We concluded that there was minimal risk of cross-contamination or carryover between wells using the automated liquid handler, and all subsequent experiments combined multiple replicates of test cases, positive controls and negative controls on individual 96-well plates. (A sample plate layout is provided in the Additional file 1: Figure S1.)

### Automated antimicrobial activity assay: planktonic culture

**Fig. 2:**
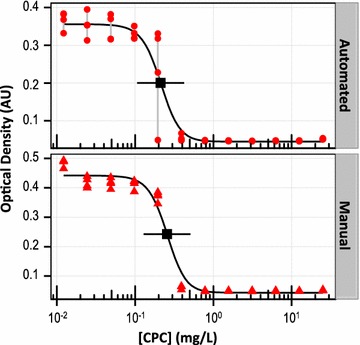
Planktonic antimicrobial activity: optical density. Antimicrobial activity assays for the known antimicrobial CPC compared favorably when performed manually (*bottom*) or with automation (*top*). Each CPC concentration was replicated four times in each assay (replicates are connected by *grey lines*). *Black traces*, *points*, and *horizontal bars* show three-factor logistic fits, calculated IC50 values, and day-to-day precision, as described in the “Materials and methods” section

The robotic liquid handler was used to automate a simple planktonic antimicrobial activity assay for the quaternary ammonium salt CPC, a known antimicrobial that is added to some commercially available mouthwashes and toothpastes. When automation was compared to manual operation (Fig. 2), the results were similar. At low [CPC], planktonic growth overnight yielded increased turbidity with no apparent antimicrobial activity, while high [CPC] prevented growth and no increase in solution turbidity was observed. The determined antimicrobial activities measured by both IC50 (0.21 mg/L = 0.61 µM for the manual assay; 0.26 mg/L = 0.75 µM for the automated assay) and MIC (0.4 mg/L = 1.2 µM for both manual and automated assays) were consistent between manual and automated assays within the precision of the methods.

Another metric for evaluating antimicrobial activity, the MBC, determines the concentration of antimicrobial compound required to completely kill bacteria (rather than just inhibiting growth). The MBC was measured by inoculating agar plates following antimicrobial exposure, thereby allowing any remaining viable bacteria to form visible colonies, and automated methods were developed (as described in the “Materials and methods” section). The measured MBC for planktonic *S. mutans* was 0.8 mg/L = 2.5 µM.

While the manual and automated procedures yielded antimicrobial activity determinations by IC50 and MIC that were indistinguishable within the measurement precision, automation was much more rapid. For all automated steps, including plate setup and inoculation as well as agar plating for MBC determination, the manual procedure required 68 min of pipetting (spread over 2 days), while the automated procedure took only 22 min (also over 2 days) and did not require the constant attention of laboratory personnel. In both cases, additional time was required for a scientist to plan the experiment (e.g., identify antimicrobial test concentrations, plate layout, and appropriate control experiments) and prepare solutions (e.g., sterile media, overnight cultures, antimicrobial stock solution). Automation additionally required time to program the instrument (estimated to be <0.5 h for this assay); however, this was a one-time investment and the program then required little modification for repeated experiments.

### Automated antimicrobial activity assay: biofilm cultures

**Fig. 3:**
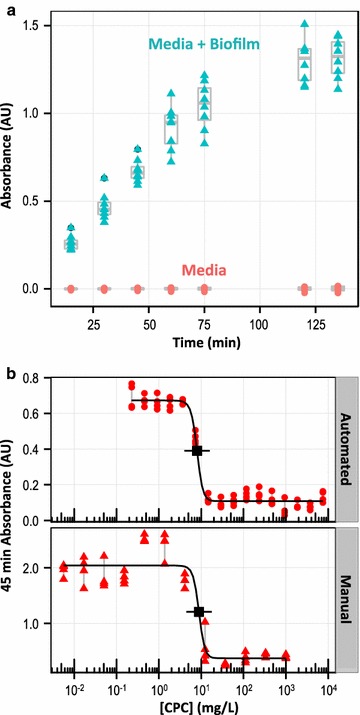
Biofilm antimicrobial activity: metabolic activity with XTT. In the presence of metabolically active *S. mutans* biofilms (i.e., no antimicrobial compound added), XTT is continuously converted to a strongly absorbing formazan derivative until depleted (**a**). Using the XTT assay response (at 45 min) to evaluate CPC antimicrobial activity against 24 h biofilms (**b**), a similar response was observed for manual (*bottom*) and automated (*top*) assays. *Grey lines* in **b** connect n = 4 replicates for each [CPC]; *black traces*, *points*, and *horizontal bars* in **b** show three-factor logistic fits, calculated IC50 values, and day-to-day precision, as described in the “Materials and methods” section

*Streptococcus mutans* biofilms were characterized by measuring their aggregate metabolic activity using the tetrazolium salt XTT, and both 24 h biofilms and biofilm forming culture conditions were amenable to this analysis approach. In the presence of metabolically active cells, XTT (non-absorbing) was converted into a strongly absorbing derivative that accumulated in solution and was readily quantified using a plate reader (Fig. 3a). After addition of XTT to the biofilm, solution absorbance increased monotonically for 100 min before plateauing (presumably due to depletion of the XTT reagent). For routine assays, biofilms were compared using specifically the absorbance at 45 min, as this provided sufficient signal sensitivity while remaining safely within the linear response range of the assay.

When 24 h *S. mutans* biofilms were exposed to varying concentrations of CPC for 3 h, antimicrobial activity was observed by measuring the biofilm metabolic activity. Similar to the results from the planktonic antimicrobial activity assay, low [CPC] conditions yielded normal highly metabolically active biofilms, while the metabolic activity of biofilms exposed to high [CPC] conditions was negligible (Fig. 3b). The magnitudes of the XTT signal are difficult to directly compare between the manual and automated methods due to inconsistent removal of media from the wells. The automated method pipetted from the middle of each well at a defined height 2 mm above the plate bottom (to minimize disruption of the biofilm) and left some media remaining in the wells. In contrast, manual operators could remove media more completely (yielding a larger XTT signal) by tipping the plate and pipetting from well corners. While the details of the methods were not optimized here (e.g., for sensitivity of response), manual and automated procedures to measure the biofilm antimicrobial activity of CPC again yielded consistent results within the precision of the measurements for IC50 (8.9 mg/L = 26 µM for the manual assay; 8.0 mg/L = 23 µM for the automated assay) or MIC (14.6 mg/L = 43 µM for both manual and automated assays).

The MBC for the 24 h biofilms was determined using similar automation to the planktonic MBC determination. However, additional steps were added when sampling the biofilm cultures to disrupt the biofilms and suspend incorporated cells with fluid shear and physical contact before agar inoculation. The whole procedure was facilitated using automation (see “Materials and methods” section), and a picture of an agar plate used for determining the MBC of 24 h biofilms is included in the Additional file 1: Figure S1. The measured 24 h biofilm MBC for CPC was 58 mg/L = 170 µM.

In addition to the protocols for assay setup and inoculation onto agar, which were similar to the planktonic assay, the analysis of biofilm activity using the XTT metabolic assay also involved multiple pipetting steps, media exchanges and timed delays that were amenable to automation. While the manual steps involved in all parts of the biofilm antimicrobial activity screen (including setup and inoculation, metabolic activity assay, and agar plating) required 115 min, the automated procedure took only 51 min of instrument time.

### CPC antimicrobial activity: planktonic, biofilm forming, and 24 h biofilm cultures

**Fig. 4:**
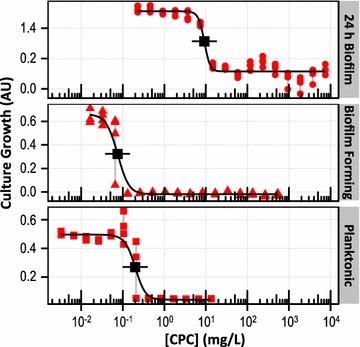
Antimicrobial activity for different culture conditions. CPC antimicrobial activity against *S. mutans* differs under conditions of planktonic (*bottom*), biofilm forming (*middle*), and 24 h biofilm (*top*) growth. Culture Growth was assessed by turbidity (for planktonic culture) or metabolic activity (for biofilm forming and 24 h biofilm cultures). *Grey lines* connect n = 4 replicates for each [CPC]; *black traces*, *points*, and *horizontal bars* show three-factor logistic fits, calculated IC50 values, and day-to-day precision, as described in the “Materials and methods” section

Having developed automated methods and validated their results against conventional manual methods, automated liquid handling was subsequently employed to increase throughput and facilitate direct comparison of the antimicrobial susceptibility of planktonic, biofilm forming, and 24 h biofilm cultures of *S. mutans* to CPC (Fig. 4). The more mature 24 h biofilms exhibited a lower sensitivity to CPC (indicated by higher IC50, MIC and MBC concentrations) than either the planktonic or biofilm forming cultures. Additionally, the process of biofilm formation exhibited slightly greater sensitivity to CPC antimicrobial activity than simple planktonic growth, possibly due to the reduced nutrient composition of the biofilm-forming media combined with the planktonic phenotype of the bacteria from overnight stock cultures used for inoculation. (The specific antimicrobial activity determinations for CPC for each culture condition can be found in Table 1. A detailed breakdown of the time required for manual or automated pipetting in each culture condition and stage of the experiment is provided in Table 2).

**Table 1:** Antimicrobial activity data

Growth condition	IC50 (µM)	MIC (µM)	MBC (µM)
Planktonic growth	CPC: 0.6	CPC: 1.2	CPC: 2.5
	CPB: 0.5	CPB: 0.6	CPB: 4.9
	CPI: 0.7	CPI: 1.2	CPI: 4.9
Biofilm forming	CPC: 0.2	CPC: 0.4	CPC: 0.8
	CPB: 0.2	CPB: 0.4	CPB: 0.8
	CPI: 0.3	CPI: 0.4	CPI: 0.8
24 h biofilm	CPC: 24	CPC: 43	CPC: 172
	CPB: 20	CPB: 43	CPB: 172
	CPI: 32	CPI: 86	CPI: 172

The antimicrobial activity of three halide salts of cetylpyridinium (CPC, CPB, and CPI) against *S. mutans* under different culture conditions (planktonic, biofilm forming, and 24 h biofilm) was measured by IC50, MIC, and MBC in a single experiment. For each condition, no significant effect was observed for the identity of the halide counter ion within the precision of these measurements

**Table 2:** Time requirements for manual and automated pipetting

Growth condition	Setup and inoculate (min)	Growth analysis (min)	Agar plating (min)
Planktonic growth	Manual: 37	Manual: 0	Manual: 31
	Automated: 16	Automated: 0	Automated: 6
Biofilm forming	Manual: 37	Manual: 31	Manual: 47
	Automated: 16	Automated: 15	Automated: 14
24 h biofilm	Manual: 16	Manual: 51	Manual: 47
	Automated: 6	Automated: 32	Automated: 14

The assays generating the data for Fig. 4 required many (nearly 3000) pipetting operations from initial set up through analysis: three culture conditions, with different media formulations and orders of operations; for each condition, sixteen antimicrobial concentrations were evaluated with four replicates and positive and negative controls; for each individual well, media, antimicrobial and cells were added in varying proportions; the metabolic activity assessment of the biofilms required multiple media exchanges; and aliquots from every well were inoculated onto agar to estimate the MBC. The time requirements (in minutes) for each automated (measured) or manual (estimated) step are tabulated here. Overall, manual pipetting would require 5 h of hands-on time while automated pipetting required only 2 h of machine time. (The actual duration of the experiments was several days for each condition to allow for bacterial growth.)

### Antimicrobial activity screening: counter ions in cetylpyridinium salts

**Fig. 5:**
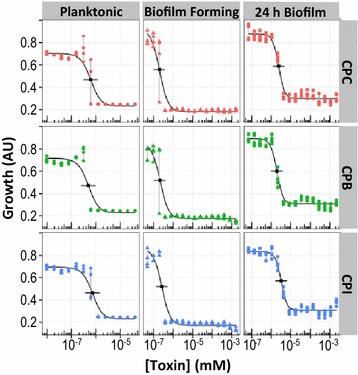
Antimicrobial activity screening. Automated methods facilitated the screening of antimicrobial activity against *S. mutans*. Planktonic (*left*), biofilm forming (*center*), and 24 h biofilm (*right*) growth conditions were evaluated for the antimicrobial activity of three halide salts (Cl^−^, Br^−^, I^−^) of the cetylpyridinium cation (labeled CPC, CPB, and CPI, respectively). Culture growth was assessed by turbidity and metabolic activity, as discussed for Fig. 4. *Grey lines* connect n = 4 replicates for each [CPC]; *black traces*, *points*, and *horizontal bars* show three-factor logistic fits, calculated IC50 values, and day-to-day precision, as described in the “Materials and methods” section. All antimicrobial concentrations were plotted as molarities to facilitate comparisons between compounds (molecular weights are listed in the “Materials and methods” section)

Cetylpyridinium salts of chloride (CPC), bromide (CPB) and iodide (CPI) were evaluated for antimicrobial activity against *S. mutans* under conditions of planktonic, biofilm forming, and 24 h biofilm growth (Fig. 5). The antimicrobial activity of each salt, for each culture condition, by several metrics, was tabulated (Table 1). When grouped by culture condition and antimicrobial activity assay, the observed variations between individual salts were all within the precision of the assays, indicating no detectable counter ion effect among the halide anions evaluated here.

## Discussion

During laboratory pipetting operations, aerosolization of solution droplets can potentially lead to contamination between wells or expose researchers to hazardous materials. This issue is a particular concern when using high throughput multi-well plate formats and working with living organisms that can expand rapidly when suitable nutrients are available. However, no carryover between wells or contamination was observed using the automated liquid handler described here (Fig. 1). The instrument had no air filtration/recirculation capability to maintain a sterile workspace and no mechanisms to sterilize equipment. However, sterile disposable pipette tips with integrated filters were used, pipetting operations were relatively rapid (as compared to manual operation), and the instrument could replace the lid over standard multi-well plates to minimize environmental exposure during delays in pipetting operations. Since no carryover or contamination between wells was detected when using the automated liquid handler, individual wells of a single 96-well plate were subsequently used to replicate experimental conditions and provide positive and negative experimental controls.

The laboratory operations required for traditional planktonic antimicrobial activity assays (e.g., the generation of suitable antimicrobial dilution series, addition of growth media, inoculating planktonic bacteria, reagent addition, agar plating) were straightforward pipetting steps and readily automated. While the manual and automated procedures produced comparable results within the measurement precision (Fig. 2), automation was much more rapid overall and did not require human intervention. Automation promises even greater timesaving when considering the entirety of a research investigation (e.g., additional assays to evaluate measurement reproducibility, increase statistical power, include more experimental controls or test additional antimicrobial compounds, bacterial strains, or media formulations). In addition to reducing the burden on laboratory staff, automation removes the potential for ‘user error’ mistakes during repetitive pipetting steps.

In addition to planktonic growth, naturally occurring bacteria commonly form biofilms on a variety of surfaces, particularly when nutrients become limited. In the oral environment, for example, it is these surface-attached biofilms that have been associated with demineralization of tooth enamel and cavity formation. Within biofilms, bacteria secrete extracellular polymeric substance (EPS), significantly change their gene expression profile, and exhibit a variety of different phenotypes, even within isogenic cultures (Kolenbrander 2000; Cvitkovitch et al. 2003; Marsh 2005; Salli and Ouwehand 2015). Unfortunately, antimicrobial materials can be significantly less effective against microbial biofilms than against planktonic cultures. Specifically, these phenotypic differences have been observed previously for CPC antimicrobial activity against planktonic and biofilm cultures of *S. mutans* under various conditions (Pallanza et al. 1984; Wilson 1996; Haps et al. 2008; Sreenivasan et al. 2013; Kitagawa et al. 2014; Latimer et al. 2015).

Here, biofilms formed spontaneously when planktonic *S. mutans* bacteria were inoculated in BFM (with decreased nutrients and excess sugar), secreting significant EPS and becoming adherent. Unlike planktonic cultures, biofilms do not generate reliable changes in turbidity that can be measured to assess growth and evaluate antimicrobial efficacy. Instead, the biofilm was characterized after 24 h by measuring its aggregate metabolic activity using the tetrazolium salt XTT, as reported previously (Koban et al. 2012). In the presence of metabolically active cells, dehydrogenase enzymes continuously converted XTT (non-absorbing) into a strongly absorbing formazan derivative (Fig. 3a). When manual and automated antimicrobial activity assays were compared for biofilms, the results were indistinguishable within the precision of the measurements (Fig. 3b). As with planktonic cultures, screening biofilms for antimicrobial activity was greatly accelerated by using automation.

Bacteria in the mouth thrive in a very dynamic environment, and attempts to manage dental health must consider the planktonic bacteria present in saliva, mature biofilms already adherent to tooth and epithelial surfaces, as well as the transitional processes of biofilm formation (e.g., following dental cleaning) and dispersal. Further, these various stages of bacterial growth may exhibit different susceptibilities to treatment with antimicrobial compounds. This is a common characteristic of microbial ecologies, complicating determination and reporting of antimicrobial activity (Marsh 2004; Harrison et al. 2004; Sabater et al. 2007; Salli and Ouwehand 2015). The use of automation in the current investigation facilitated side-by-side comparison of three distinct phenotypes (i.e., planktonic, biofilm forming, and 24 h biofilm cultures) by significantly improving experimental throughput as compared to manual operation (Fig. 4). Directly compared side-by-side, the sensitivity of biofilm forming cultures to CPC antimicrobial activity was much greater than 24 h biofilm cultures and even slightly greater than planktonic cultures.

By fully automating the assays, the time required to setup, inoculate and analyze antimicrobial activity against multiple culture phenotypes was significantly reduced (Table 2). Once the automated liquid handler had been programed and validated (i.e., Figs. 2 and 3), minimal time (≤10 min) was required to load and reuse the existing procedures (i.e., Fig. 4). For all of the data used in Fig. 4 to compare the antimicrobial sensitivities of planktonic, biofilm forming, and 24 h biofilm phenotypes, only 2 h of machine time was involved in automated pipetting, while manual operation would have required 5 h of hands-on pipetting. When considering the staffing hours that are saved by using an automated liquid handler, the upfront resource requirements (instrument acquisition and training) can be properly accounted for. Automation additionally reduced the number of opportunities for human error to arise (e.g., addressing the wrong well of a plate) during the many (nearly 3000) repetitive operations required just for Fig. 4. Although some operations are not currently amenable to automation (i.e., only relatively simple pipetting operations were evaluated here), robotic capabilities have been advancing rapidly.

There has been disagreement in the literature about the role that counter ions may play in the antimicrobial activity of quaternary ammonium salts (Shelton et al. 1946; Panarin et al. 1985; Kanazawa et al. 1993; Chen et al. 2000). Exhaustive screening of closely related compounds within a single lab can be arduous to perform manually, and reports from different laboratories are difficult to compare. In contrast, the automated protocols reported here were easily extended to screen a panel of antimicrobials (for several microbial phenotypes) since the methods had already been developed. The results depicted in Fig. 5 and Table 1 (no detectable counter ion effect for the halide anions evaluated) would have required an estimated 15 h of hands-on pipetting had they been performed manually.

Overall, automation was successfully employed to expedite routine antimicrobial activity screening of compounds against microbial cultures. Protocols for the automated preparation of planktonic, biofilm, and agar colony cultures of *S. mutans* were developed and validated. Coupled with high-throughput OD/absorbance measurements, planktonic, biofilm forming, and 24 h biofilm antimicrobial activity assays were automated for the known antimicrobial CPC and compared favorably with manual antimicrobial activity assays by several common metrics (e.g., IC50, MIC, and MBC). In addition to minimizing the potential for ‘human error’ (e.g., pipetting from the wrong well), automation greatly increased throughput by decreasing the amount of time and user intervention required for repeated assays. Using automated methods, a series of cetylpyridinium salts were screened and exhibited no detectable counter ion effect on their antimicrobial activity against *S. mutans*. As greater attention is focused on screening potential antimicrobial compounds against a range of microbial strains and culture conditions, automation promises to increase throughput, minimize expense and ensure assay reproducibility.

## Additional file

10.1186/s13568-016-0191-2 Supplementary information is available from the publisher and includes a 96-well plate experimental layout and an example of agar plating from biofilm cultures for MBC assessment. All raw data and analysis code are publically available (Forry 2015), and automation methods are available upon request.

## Footnotes

1 Certain commercial equipment, instruments, and materials are identified in this paper to describe the experiments performed. Such identification does not imply recommendation or endorsement by the National Institute of Standards and Technology, nor does it imply that the materials or equipment identified are necessarily the best available for the purpose.
