# Supplementary material for: Automation of antimicrobial activity screening
Source: AMB Express. 2016 Mar 12;6:20. doi: 10.1186/s13568-016-0191-2 (PMC4788993; doi:10.1186/s13568-016-0191-2)
Supplement: Supplementary file 1 — 10.1186/s13568-016-0191-2 Supplementary information is available from the publisher and includes a 96-well plate experimental layout and an example of agar plating from biofilm cultures for MBC assessment. All raw data and analysis code are publically available (Forry 2015), and automation methods are available upon request. [file 13568_2016_191_MOESM1_ESM.pdf]

AMB Express

Automation of Antimicrobial Activity Screening: Supplementary Information

Samuel P. Forry,[*1] Megan C. Madonna,[1] Daneli López-Pérez, [1,2]
Nancy J. Lin, [1] and Madeleine D. Pasco[1]

[*] Corresponding Author: sforry@nist.gov

[1] Biosystems and Biomaterials Division, National Institutes of Standard and Technology, Gaithersburg, MD, 20899.

[2] Currently at the Center for Drug Evaluation and Research, Food and Drug Administration, Silver Spring, MD, 20993.

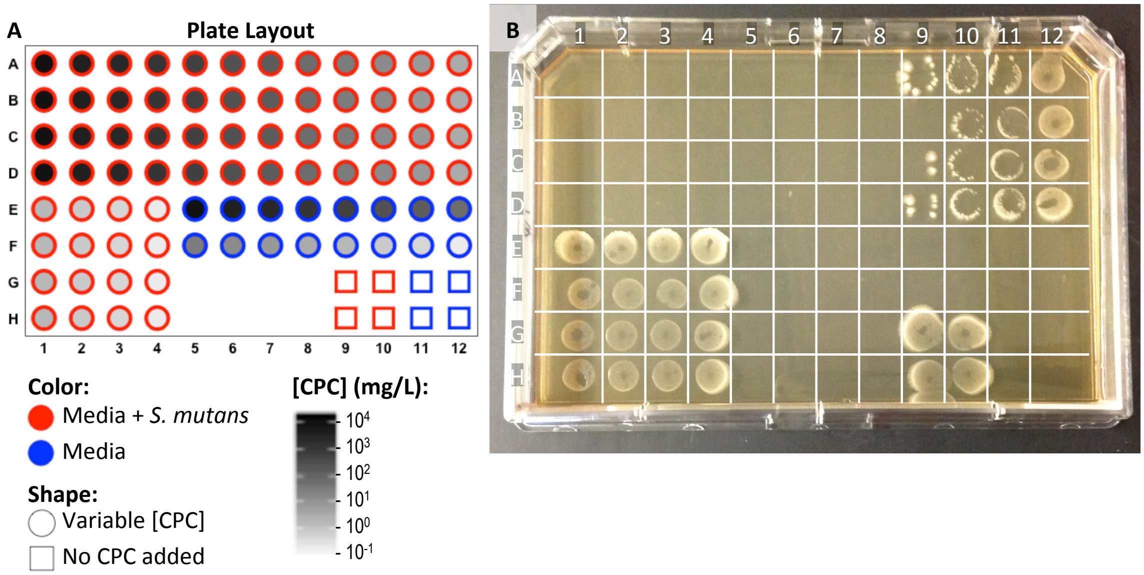

Figure S1: Plate layout and agar plating. The antimicrobial assays presented were performed in 96-well plates that included at least 4 replicates for each test condition (e.g., antimicrobial concentration) as well as positive controls (e.g., bacteria in the absence of antimicrobials) and negative controls (e.g., antimicrobial in the absence of bacteria or media alone). The plate layout (A) is shown with the color outline indicating wells where *S. mutans* were seeded, and the shape indicating variable antimicrobial concentration (circles, with discrete concentrations coded in the grey centers) or control wells without antimicrobial (squares). The discrete concentrations of antimicrobial used were 2-fold serial dilutions; the actual concentrations shown here were used for the 24 h biofilm phenotype.

Following growth assessment, aliquots from each culture well of the antimicrobial assays were plated onto agar in a 1-well plate and incubated for 48 h (B). The resulting growth of colonies indicated the presence of residual viable bacteria after antimicrobial treatment. Since only 5 μl of the cell suspensions were transferred, surface tension prevented the discrete aliquots from drifting or merging on the agar surface. The positional precision of the automated liquid handling robot ensured that the location of each sample correlated with its address on the originating 96-well plate. (The white numbers, letters, and grid lines in B were superimposed to facilitate data interpretation when compared to the plate layout shown in A.) As seen here for CPC antimicrobial activity against 24 h biofilms, colony growth was evident at locations correlating with wells containing low antimicrobial concentrations or positive experimental controls (i.e., for wells A9-D12, A1-H4, and G9-H10), and the MBC identified was 58 mg/l.
